# Different zeolite systems for colon cancer therapy: monitoring of ion release, cytotoxicity and drug release behavior

**DOI:** 10.1007/s40204-019-0115-8

**Published:** 2019-05-20

**Authors:** A. G. Abd-Elsatar, M. M. Farag, H. F. Youssef, S. A. Salih, M. M. Mounier, E. El-Meliegy

**Affiliations:** 1 Ceramics, Refractories and Building Materials Department, National Research Centre, 33 El-Buhouth Street, Dokki, Cairo, 12622 Egypt; 2 Glass Research Department, National Research Centre, 33 El-Buhouth Street, Dokki, Cairo, 12622 Egypt; 3 Physical Chemistry, Faculty of Science, Cairo University, Cairo, Egypt; 4 Pharmacognosy Department, Pharmaceutical and Drug Industries Research Division, National Research Centre, El-Buhouth Street, Dokki, Cairo, 12622 Egypt

**Keywords:** Fluorouracil (5-Fu), Drug delivery, Synthetic zeolites, Simulated gastric fluid, Colon cancer

## Abstract

**Abstract:**

Three types of oral administrated micronized zeolites [ZSM-5, zeolite A and Faujasite NaX (ZSM-5, ZA and ZX, respectively)] were prepared as anticancer 5-fluorouracil (5-Fu) delivery systems for colon cancer treatment. They were prepared by economically widespread and cheap natural resource, kaolin, at low temperatures, using microwave advanced tool. The obtained powders were characterized by XRD, SEM/EDX and BET; meanwhile, their degradation was investigated in two gastric fluids; FaSSGF (pH 1.6) and FeSSGF (pH 5), through concentration measurement of their solution disintegrated elemental constituents of Na^+^, Al^3+^ and Si^4+^ ions. Also, the processes of drug release and mechanism in both solutions were investigated. Moreover, the inhibition action of 5-Fu-free and 5-Fu-conjugated zeolites on colon cancer cells (CaCo-2) was estimated. The results showed that, the prepared zeolites possessed high surface areas of 526, 250, and 578 m^2^/g for ZSM-5, ZA and ZX, respectively. Although, zeolite structures seemed significantly stable, their frameworks seemed more likely reactive with time. The ions and drug release for zeolites occurred in successively two stages and found to be pH dependent, where the drug and zeolite ions were significantly of higher values in the more acidic media of the gastric solution (pH 1.6) than those of the mild acidic one (pH 5). The obtained activity indicated no cytotoxic affinity for all the prepared zeolite types. Accordingly, the synthesized zeolite frameworks are proposed to be of strong potential drug delivery vehicle for the treatment of gastrointestinal cancer.

**Graphical abstract:**

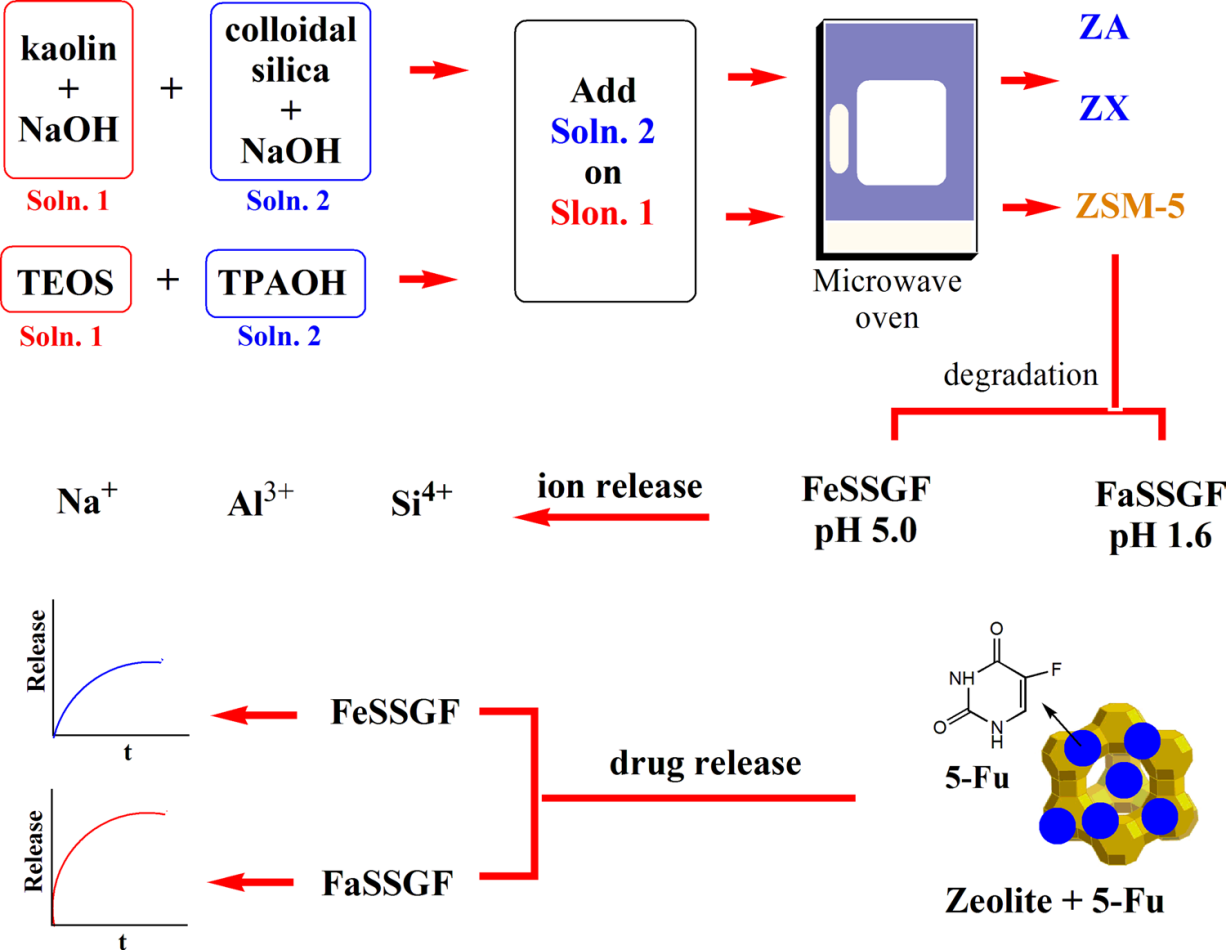

## Introduction

Colon cancer is recorded as the third cancer that causes the death in the world (Banerjee et al. 2017). There are three main ways for treating this cancer type: surgery, chemotherapy and radiation. Chemotherapy is almost applied after surgery, but, in some cases it is used before surgery to reduce and constrict the cancer, followed by using drugs to destroy cancer cells. However, toxicity and denaturing in gastrointestinal system are almost the problems of conventional drugs associated with chemotherapy. Therefore, loading of the drug on biocompatible carriers able to stand in the digestive system for longer time is considered a versatile method to deliver the drug to targeted diseased tissue. Among these carriers are zeolites which showed an oral drug sustained release vehicle (Sanders 1990).

Zeolites are hydrated microporous crystalline aluminosilicates that build in a three-dimensional open network ring structures of SiO_4_ and AlO_4_ tetrahedra. This unique structure of zeolites makes them ideal matrices for encapsulating ions, nanoparticles and drugs, with the ability of their release in a sustained manner. For such instance, several studies proved the convenience of zeolites as biocompatible and non-toxic materials for different kinds of cells (Kralj and Pavelic 2003; Thom et al. 2003; Thomassen et al. 2012). Hence, they are considered as excellent pharmaceutical candidates for drug delivery systems (Youssef et al. 2008). Definitely, they are used successfully as a carrier for various drug molecules for chemotherapeutic treatment of cancers (Rimoli et al. 2008; Pasquino et al. 2016; Khodaverdi et al. 2016). On the other hand, the economic synthesis of high-purity zeolites, used in such application, can be made from cheap widespread refined raw materials (e.g., kaolin rock) with reduced energy consumption.

Fluorouracil (5-Fu) has possessed more attention as an anticancer drug. It is a water-soluble pyrimidine analog, which is one of the most effective chemotherapeutic agents and cytotoxic agents in the treatment of the gastrointestinal cancers. This drug can be delivered in oral, topical, and aerosolized formulations. The inhibition of thymidylate synthase and the incorporation of its metabolites into RNA and DNA are the main function of 5-Fu as an anticancer drug (Datt et al. 2013; Spanakis et al. 2014; Vilaça et al. 2013; Al-Thawabeia and Hodali 2015; Sağir et al. 2016). Several previous studies used zeolites to encapsulate 5-Fu. HY zeolite was inspected in biological fluids under different physiological conditions (Datt et al. 2013), zeolite X and BEA were estimated as anticancer drug delivery system through the oral route of administration (Spanakis et al. 2014), and zeolite L and Y against colorectal carcinoma, one of the most common types of cancer in industrialized countries (Vilaça et al. 2013). Also, the silicate zeolites such as ZSM-5 and H-ZSM-5 were applied as eminent carriers for 5-Fu (Al-Thawabeia and Hodali 2015). Furthermore, magnetite–zeolite nanocomposite particles were also tested as 5-Fu carrier with drug loading, releasing capacity and biological activities in gastric cancer cell line AGS (Sağir et al. 2016).

When we talk about zeolite as a drug carrier for colon cancer, it is important to discuss zeolite solubility in solutions with different pH values, because this carrier passes through different pH regions of the digestive system up to the colon. Accordingly, several studies investigated the drug release profiles from the zeolite at different pH conditions to mimic gastrointestinal fluids (Rimoli et al. 2008; Khodaverdi et al. 2016; Karavasili et al. 2017). However, most of the previous studies, if not all, used simulated fluids similar to the intestinal ones in terms of pH value only (such as HCl, acetate buffer and phosphate buffer saline solutions), and not in terms of the chemical composition, as well. Therefore, there is a need to study, not only the drug release profile in simulated intestinal fluids with respect to pH, but also as a function of chemical compositions of analog fluids. In addition, there are no intensive studies investigating the dissolution of zeolite vehicle during drug release, as well.

To the best of our knowledge, this is the first paper aiming at giving an economic value to the carrier (zeolite) by its synthesis from inexpensive and widespread raw material (kaolin rock) and by efficient preparation, at reduced formation energy and high purity, using microwaves advanced tool. Three types of micronized zeolites; ZSM-5, Zeolite A and Faujasite NaX, were prepared in this study and loaded with anticancer drug (5-Fu), to be used as delivery systems for oral administration. The drug release behavior in simulated gastric fluid (SGF) and the direct effect of zeolite immersion on the instant release of ions from zeolites surfaces were investigated. Furthermore, cytotoxicity of zeolites with/without 5-Fu on the precultured colon cancer cells was also tested.

## Materials and methods

### Materials

Refined kaolin, supplied by Middle East Mining Company (MEMCO), Egypt, was used as the starting material for zeolite formation. The chemical composition (wt%) of the calcined kaolin (meta-kaolinite), as given by the XRF (X-ray fluorescence) analysis, contained 53.25 SiO_2_, 42.94 Al_2_O_3_, 0.41 Fe_2_O_3_, 1.62 TiO_2_ and some other minor constituents of MgO, CaO, and K_2_O. Kaolin is commonly used as a starting material for preparing zeolite-A since its Si/Al ratio is near unity as that of zeolite-A (Chandrasekhar et al. 1997; Bougeard et al. 2000). Tetraethyl orthosilicate (TEOS) 98% (Sigma-Aldrich), tetrapropyl ammonium bromide (TPABr) 98% (Sigma-Aldrich) and sodium hydroxide pellets (NaOH) with the composition of 98.6% NaOH (Sigma-Aldrich) were used as pure chemicals. Ludox AS-40 colloidal silica, 40 wt% suspension (Sigma-Aldrich), was utilized as the additional SiO_2_ source for adjusting the kaolin composition to match that of the targeted zeolite X Faujasite.

### Preparation of different zeolites

In the present study, three zeolite types were taken care of: two of aluminosilicate composition (Zeolite-A (ZA), and Faujasite-NaX (ZX)) along with a completely silicate type of silicalite structure (ZSM-5).The ZSM-5 was prepared by placing 18.34 g tetrapropyl ammonium bromide in a Teflon container under vigorous stirring (800 rpm). During stirring, about 15 mL of 0.01 M NaOH was added and the mix was stirred for 1 h. An amount of 20.83 g tetraethyl orthosilicate was then dropwise added to the previous solution. The mixture was stirred for another 30 min and an amount of 18.57 g distilled water was finally added; the whole slurry was kept at room temperature for 24 h under vigorous stirring. 15–20 mL of the previous transparent solution was then loaded to the Xpress vessels of the microwave (MARS Extraction and Digestion system, Model XP-1500, CEM Corp., Matthews, NC). This microwave system operated at a frequency of 2.45 GHz and can proceed from 1 to 100% of 1600 W power. Precursor gel of ZSM-5 was heated at 140 °C for 5 h under M–H at 400 W and a pressure of 20 psi. The synthesis product was then collected, washed, several times, with distilled water on centrifugation, and finally dried overnight at 100 °C.

Zeolite A (encoded ZA) was prepared as described before using microwave irradiation at 80 °C for 2 h (Youssef et al. 2008). Meta-kaolinite, produced by calcinations of Egyptian kaolin at 700 °C for 4 h, and 3.0 M NaOH solution were used as starting materials. The solid/liquid ratio of meta-kaolinite to alkaline solution was 1.0 g/25 mL. The slurry was transformed to the microwave instrument for treatment at 80 °C for 2 h, and then it was centrifuged and the solid was washed several times with deionized water to remove the excess alkalinity and air-dried at 60 °C overnight.

Faujasite-NaX (encoded ZX) was prepared based on its Si/Al ratio. 3.0 M NaOH solution was divided in two Teflon vessels with a capacity of 100 mL. Meta-kaolinite was added to one of the two vessels with the liquid/solid ratio of 12 (solution 1); whereas, 40 mL of colloidal silica was reacted with the second alkali solution in another vessel (solution 2). Solution 1 was then added to solution 2 dropwise during vigorous stirring. The whole slurry was then stirred and transferred to the vessels of the microwave instrument to be treated at 110 °C for 2 h, and finally it was centrifuged and washed following the above-mentioned steps.

### Zeolite characterization

The mineralogical constituents of the prepared zeolites was investigated by x-ray diffraction method, using BRUKUR D 8 ADVANE with secondary monochromatic beam CuK radiation at keV = 40 and mA = 40. Microstructures of the prepared materials were studied using SEM (Quanta 250 FEG, Field Emission Gun) attached with EDX Unit, with accelerating voltage 30 kV(FEI, Netherlands). The specific surface area, pore and particle size distribution of the prepared zeolites were determined from nitrogen adsorption–desorption isotherms using NOVA 2000 series, chromatic, UK at 77 K. The Barrett–Emmett–Teller (BET) method was utilized to calculate the specific surface areas. The total pore volume was derived from the adsorption branches of the isotherms using the Barrett–Joyner–Halenda (BJH) method. The total pore volume was estimated from the amount adsorbed at a maximum relative pressure.

### In vitro dissolution test in FaSSGF and FeSSGF

In this test, the zeolites samples were tested in a fast state of the simulated gastric fluid (FaSSGF) and fed-state simulated gastric fluids (FeSSGF) prepared according to previous procedures (Marques et al. 2011; Vellaian Karuppiah and Manavalan 2012), and their compositions are illustrated in Table 1. Briefly, FaSSGF (pH 1.6) was prepared by dissolving 0.16 g of lecithin in 1.6 mL of dichloromethane, and 0.42 g of sodium taurocholate in 5 L of distilled water. Then, 1.0 g pepsin and 20 g of NaCl were added and the mixture was heated to 40 °C, and the volume was set to 10 L by distilled water (Vellaian Karuppiah and Manavalan 2012). FeSSGF was prepared by dissolving 138.5 g NaCl and 40.04 g sodium acetate in 5 L water, and 10 mL of acetic acid was added and diluted to 10 L with water (acetate buffer). Then, fully fat milk with 1:1 in percentage of the acetate buffer was added (Vellaian Karuppiah and Manavalan 2012).

**Table 1:** Composition of the simulation medium in simulated fast-state gastric fluid (FaSSGF) fed-state simulated gastric fluid (FeSSGF)

	FaSSGF	FeSSGF
Composition		
Sodium taurocholate (μM)	80	–
Lecithin (μM)	20	–
Pepsin (mg/mL)	0.1	–
Sodium chloride (mM)	34.2	237.2
Acetic acid (mM)	–	17.12
Sodium acetate (mM)	–	29.75
Milk/acetate buffer	–	1:1
Hydrochloric acid q.s. pH	1.6	5.0
Properties		
pH	1.6	5.0
Osmolality (mOsm/kg)	120.7 ± 2.5	400
Buffer capacity (mmol/L/pH)	–	25
Surface tension (mN/m)	42.6	–

The dissolution test was carried out by immersing 0.2 g of sample in 45 mL of FaSSGF or FeSSGF and incubated at 37 °C. At predetermined times (from 1 to 96 h), 10 mL of solution was separated and replaced by fresh one, and pH of such solutions was measured. Na^+^, Al^3+^ and Si^4+^ ion concentrations were measured by ICP-OES (Agilent 5100 Synchronous Vertical Dual View).

### Drug delivery test

5-Fu was loaded by immersing 45 mg of sample in 10 mL of drug solution (50 mg/L) in falcon tube and incubated at 37 °C for 2 days to allow the adsorption of drug on the surface of the powder samples. After that, the powders were separated by centrifuging the sample tubes, and the filtrate was removed and the powder was completely dried in the incubator. The drug adsorbed by the zeolite samples was calculated as the difference in drug concentration before and after powder immersion. The amounts of 5-Fu were measured by UV–Vis spectrometer (model, SP-2000UV) at a wavelength of 266 nm (Santos et al. 2009; El-Ghannam et al. 2010), and the adsorbed amount of drug was determined from previously established standard curve.

The drug release was examined in FaSSGF or FeSSGF. FeSSGF was prepared without adding milk to avoid interference during drug concentration measurement. The drug-loaded powders were soaked in FaSSGF or FeSSGF and incubated at 37 °C. Thereafter, at predetermined times (from 10 to 720 min.), 2 mL of supernatants was separated, kept at − 4 °C and replaced by fresh 2 mL. The amounts of released drug were determined as mentioned above.

Drug release mechanism of different samples investigated by fitting of different release data in the following kinetic models:

Higuchi model: it is the relation between cumulative percentage of released drug and square root of time (Higuchi 1963). This model is useful for studying the release of water-soluble and poorly soluble drugs from a variety of matrices, including solids and semi-solids.

1 $$Q_{t} = K_{{\text{H}}} \times t^{{1/2}} .$$

Baker–Lonsdale model (Costa and Lobo 2001): this model was developed from the Higuchi model and described the drug release from spherical matrices according to the equation:

2 $$3/2\left[ {1 - \left( {1 - Q_{t} } \right)^{{2/3}} } \right] - Q_{t} = K_{{{\text{BL}}}} t,$$

where *Q*_*t*_ is the amount of drug released in time *t*, *Q* the initial amount of drug in the sample, and *K*_H_ and *K*_BL_ are rate constants calculated from Higuchi and Baker–Lonsdale models, respectively. The regression coefficient, *R*^2^, was used as an indication of data fitting to know the mechanism of drug release from these formulations.

### Antitumor activity test

Cytotoxicity of drug-free and conjugated drug samples was tested using human colorectal adenocarcinoma cells (Caco-2 cell line) which were obtained from Karolinska Center, Department of Oncology and Pathology, Karolinska Institute and Hospital, Stockholm, Sweden. The procedure was performed in a sterile area using a laminar air flow cabinet biosafety class II level. Culture was maintained in DMEM medium with 1% antibiotic–antimycotic mixture (10,000 U/mL potassium penicillin, 10,000 μg/mL streptomycin sulfate and 25 μg/mL amphotericin B), 1% l-glutamine, and supplemented with 10% heat inactivated fetal bovine serum (Thabrew et al. 1997). Doxorubicin was used as a positive control. A negative control composed of DMSO was also used.

Following, the cells were cultured for 10 days; cell cytotoxicity assay was carried out according to Sathupunya et al. (2004). Briefly, the cells were seeded at a concentration of 20 × 10^3^ cells per well in a fresh complete growth medium using 96-well microtiter plastic plates at 37 °C for 24 h under 5% CO_2_, in a water-jacketed carbon dioxide incubator. Fresh medium (without serum) was added and cells were incubated either alone (negative control) or with samples to give a final concentration of 100 μg/ml. After 48 h of incubation, the medium was aspirated and then 40 μL MTT salt (2.5 mg/mL) was added to each well and incubated for further 4 h at 37 °C under 5% CO_2_. Isopropyl alcohol was added to each well and the absorbance was measured using a microplate multi-well reader at 595 nm and a reference wavelength of 690 nm. Cell cytotoxicity was assessed according to the mitochondrial-dependent reduction of yellow MTT (3-(4, 5-dimethylthiazol-2-yl)-2, 5-diphenyltetrazolium bromide) to purple formazan.

### Statistical analyses

All experimental data stated in this work were expressed as the average ± standard deviation (SD) for *n* = 3 and were analyzed using standard analysis of Student’s *t* test. The level of significance (*P* value) is set at < 0.05.

## Results and discussion

### Characterization results of zeolites

#### XRD for mineralogical detection

**Fig. 1:**
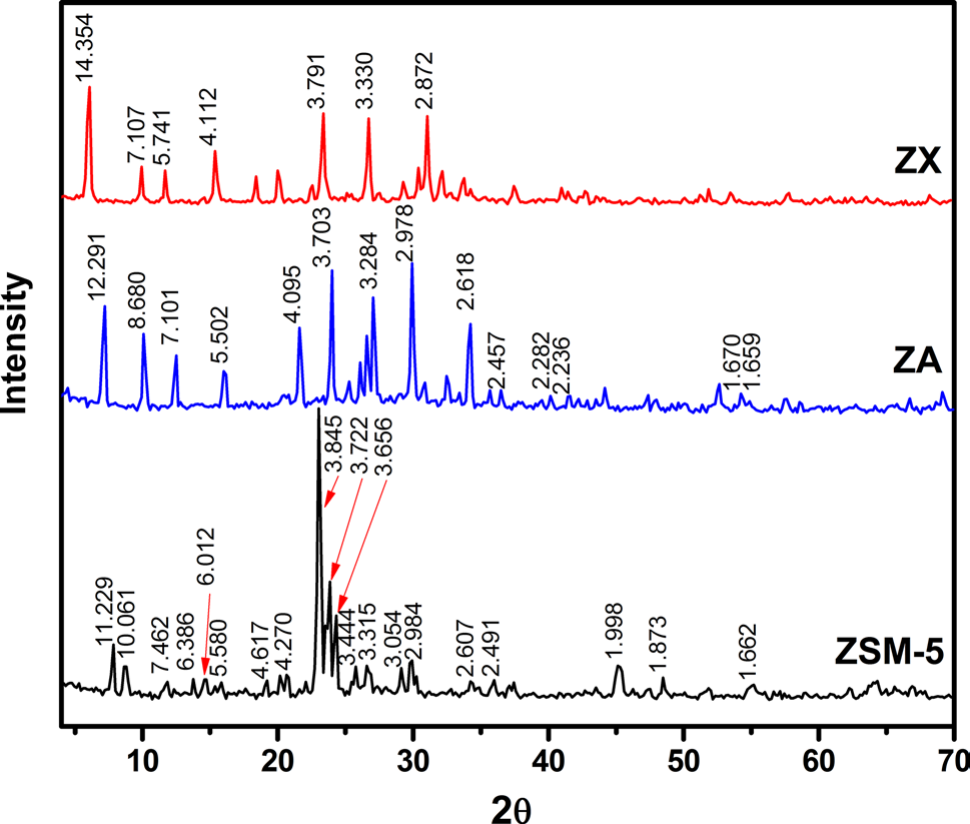
XRD patterns with d-spacing values of different zeolites hydrothermally heated at different temperatures and durations via microwave technique

Figure 1 illustrates the XRD patterns of micronized zeolites, ZSM-5, ZA and ZX, prepared under microwave conditions of 140, 80 and 110 °C for 5, 2 and 2 h, respectively. The obtained XRD peak positions and intensities were matched well with the reference cards of ZSM-5 (PDF # 43-0322(Q), linde-type A zeolite (PDF # 73-2340) and Faujasite-Nax (PDF#12-0228) with corresponding chemical compositions of Na_2.2_Al_2_Si_91_O_186.1_, Na_12_Al_12_Si_12_O_48_.27H_2_O and Na_2_Al_2_Si_4_O_12_.8H_2_O, respectively. The sharp and complete sets of peaks with high intensities indicated the formation of well crystalline powder with nearly complete crystallization, since there are no background humps. The chemical composition given by the XRD data reflected the aluminum contents of each zeolite type with a successive rational Si/Al order of ZA > ZX ≫ ZSM-5.

#### SEM–EDX analysis

**Fig. 2:**
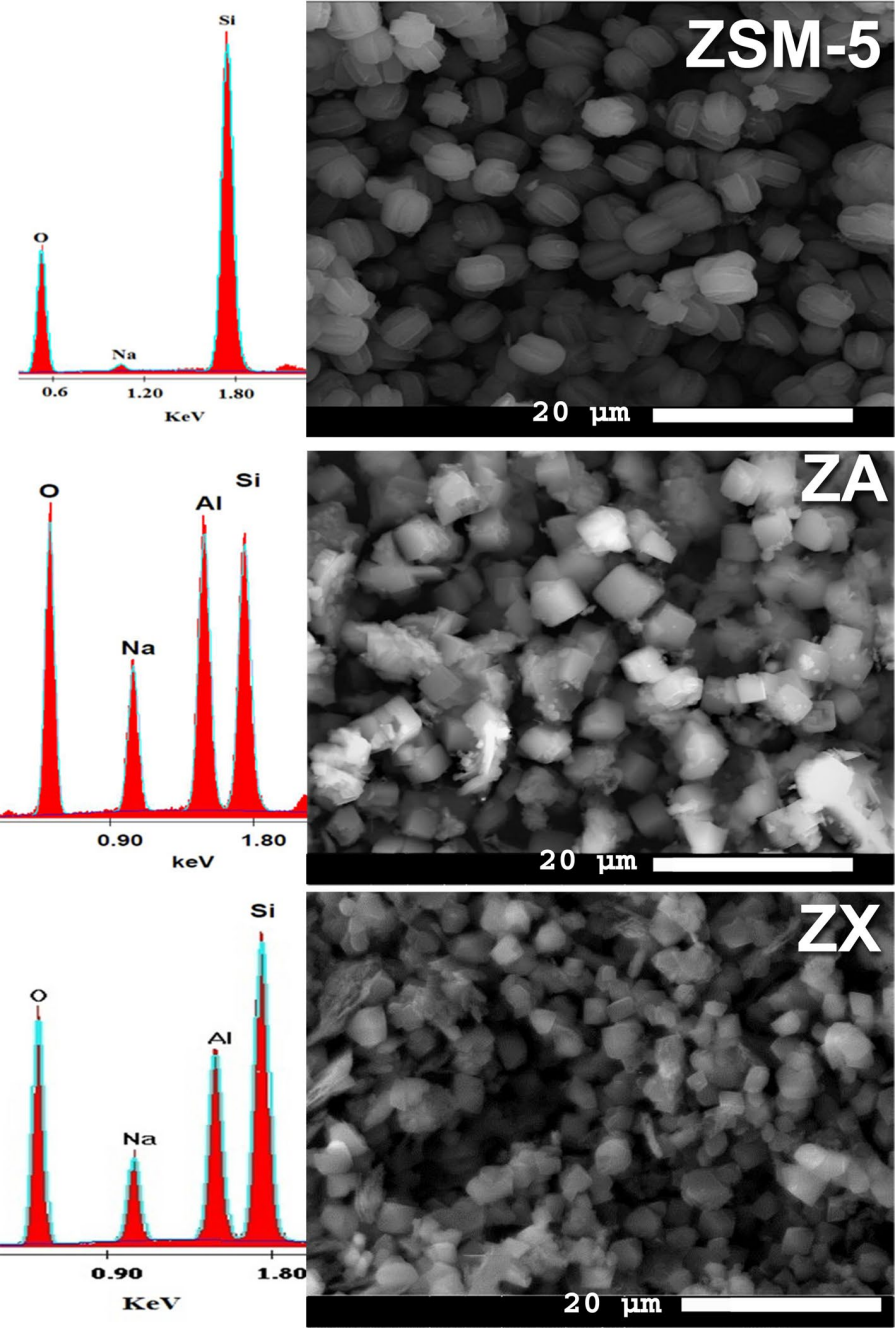
SEM micrographs and EDX analysis for ZSM-5, ZA and ZX samples

SEM micrographs and EDX analysis of the zeolites (ZSM-5, ZA and ZX) are shown in Fig. 2 and Table 2. The average crystallite sizes of zeolite powders were found to be less than 7 µm. The microstructure indicated well-developed zeolite phases with interlocked twinning plate-shaped ZSM-5 crystals, cubic ZA and bipyramidal-shaped particles of ZX. For each zeolite type, particles were uniform in size with narrow particle size distribution of 6–7, 4–5 and 2–3 µm for ZSM-5, ZA and ZX, respectively. This uniform structure of the prepared powders was due to the fact that microwave heating was volumetric and penetrating through and distributed in a fast and homogenous way to affect reactants, and favoring earlier and simultaneous nucleation of minute crystals with narrow particle size distribution. Energy saving, cost-effective and time reduction were the main benefits of using microwaves in hydrothermal synthesis (Youssef et al. 2008; Sathupunya et al. 2004).

**Table 2:** EDX elemental in atomic (at)%, surface area (m^2^/g) and total pore volume (cc/g) of ZSM-5, ZA and ZX

	EDX analysis (at%)				Texture analysis	
	O	Na	Al	Si	Surface area (m^2^/g)	Total pore volume (cc/g)
ZSM-5	52	0.91	0.4	46.69	526	1.34 × 10^−2^
ZA	51.3	12.1	17.2	19.2	251	0.78 × 10^−2^
ZX	56.1	8.7	12.8	22.4	578	2.86 × 10^−2^

EDX analysis displayed in Table 3 implies different atomic percentages (at%) of zeolites constituents, which in turn, indicated different Si/Al ratios for ZA (1.12) and ZX (1.75), since ZSM-5 has a neglecting amount of aluminum per unit cell (~ 0.4). The results obtained from the EDX analyses for zeolite samples well agreed with the previous data given by XRD cards. ZA contained much more Na^+^ percentage (12.1%) than that detected for ZX (8.7%) and ZSM-5 (0.91%), while Al contents were found in the respective order of ZA > ZX ≫ ZSM-5; thus, the latter percentage of Al can be neglected.

**Table 3:** The release rates (ppm h^−0.5^) of Si, Na and Al ions from the samples ZSM-5, ZA and ZX in FaSSGF and FeSSGF (Stage I: 1–12 h and Stage II: 12–96 h)

Ion	Sample	FaSSGF		FeSSGF	
		Stage I	Stage II	Stage I	Stage II
Na	ZSM-5	1885.5	96.5	1243.9	252.3
	ZA	1859.4	109.8	1298.1	244.5
	ZX	1819.4	105.2	1262.6	215.3
Al	ZA	37.4	23.9	15.6	1.4
	ZX	33.5	19.3	21.7	0.9
Si	ZSM-5	0.26	0.06	0.7	0.33
	ZA	16.07	10.8	5.7	2.87
	ZX	23.08	8.7	8.9	3.03

#### Surface area

The surface area (m^2^/g) and total pore volume (cc/g) of ZSM-5, ZA and ZX samples are shown in Table 4. The measured surface areas were about 526, 251, and 578 m^2^/g for ZSM-5, ZA and ZX, respectively. As seen from the table, ZX has the highest value of BET surface area of 578 m^2^/g and the total pore volume of 2.86 × 10^−2^ cc/g, whereas ZA has the lowest with a corresponding value of 251 m^2^/g and 0.78 × 10^−2^ cc/g, respectively. The previous result agreed well with the fact that ZX is a member in the Faujasite zeolite group of minerals that has the widest channel diameter of 7.4 Å, compared to 4.2 and 4–6 Å for zeolite-A and ZSM-5, respectively (Mumpton 1999; First et al. 2011). Also, it was not surprising for ZX to show the largest BET value, since it had the smallest crystallite sizes (2–3 µm), compared to ZA (5–6 µm) and ZSM-5 (6–7 µm) types.

**Table 4:** Regression coefficients (*R*^2^) and two release rates, *K*_H_ and *K*_BL_, calculated from Higuchi and Baker–Lonsdale models, respectively

		Higuchi				Baker–Lonsdale	
		Stage I		Stage II			
		*R* ^2^	*K*_H_	*R* ^2^	*K*_H_	*R* ^2^	*K*_BL_
FaSSGF	ZSM-5	0.945	5.3	–	–	0.991	11 × 10^−4^
	ZA	0.952	10.8	–	–	0.983	47 × 10^−4^
	ZX	0.971	9.2	–	–	0.993	19 × 10^−4^
FeSSGF	ZSM-5	0.908	7.0	0.949	1.7	0.972	4 × 10^−4^
	ZA	0.894	7.6	0.995	1.4	0.889	5 × 10^−4^
	ZX	0.918	9.7	0.986	1.7	0.933	7 × 10^−4^

### In vitro zeolite dissolution test in FaSSGF and FeSSGF

During the whole span of experiments (1–96 h), zeolites showed different behavior in liberating their ions into the two immersing solutions. Two main stages were noticed; stage I (0–12 h), with the relatively rapid ion release, and the later stage II (starting 12 h and afterwards) within which a very slow discharge of elements into the soaking media occurred. The release rate of each stage was calculated by fitting the ion concentration with square root of time (h^−0.5^).

**Fig. 3:**
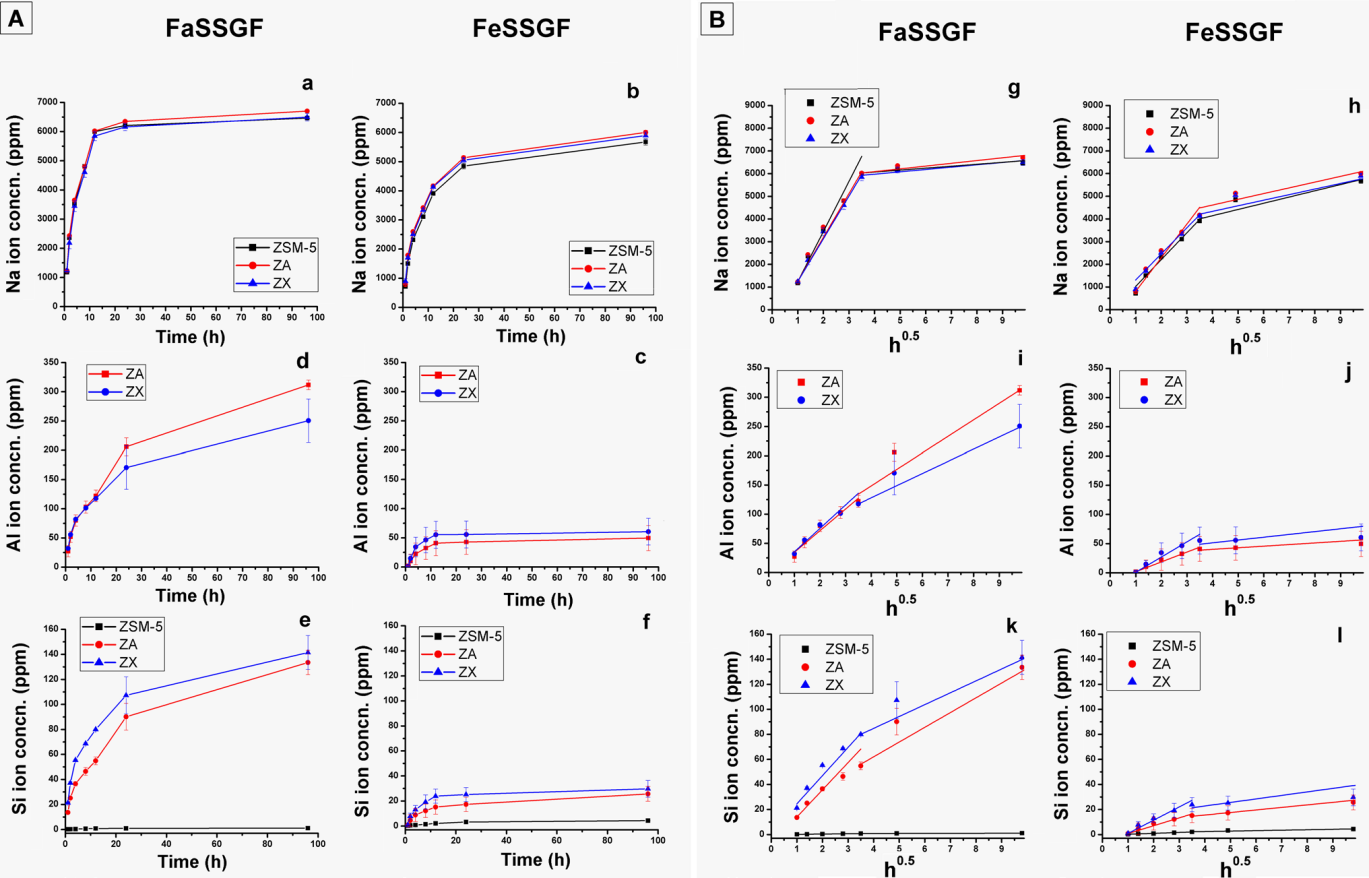
**a** Cumulative concentration of Na^+^ (a and b), Al^3+^ (c and d) and Si^4+^ (e and f) ions released from ZSM-5, ZA and ZX, and **b** cumulative concentration fitted with h^−0.5^ for Na^+^ (g and h), Al^3+^ (i and j) and Si^4+^ (k and l) ions released from ZSM-5, ZA and ZX after soaking in FaSSGF and FeSSGF, respectively

Figure 3a and b shows the Na^+^ ions released in FaSSGF(pH 1.6) and FeSSGF (pH 5) solutions with time. In stage I and for the respective type of ZSM-5, ZA and ZX zeolites, the liberated Na^+^ ions in FaSSGF(1885.5, 1859.4 and 1819.4 ppm h^−0.5^) were notably higher than those eluted in the FeSSGF (1243.9, 1298.1 and 1262.6 ppm h^−0.5^) solution. In stage II and for the same sequence of zeolites, a somewhat reduced rates of sodium ions prevailed in both solutions; FaSSGF (96.5, 109.8 and 105.2 ppm h^−0.5^) and in FeSSGF (252.3, 244.5 and 215.3 ppm h^−0.5^). Table 3 shows the whole data. Considering the values of freed sodium ions, there were no significant differences between the concentrations of Na^+^ ions detached from each zeolite type in both solutions. This finding indicated that the liberation of Na^+^ ions from zeolites did not depend on the zeolite type, but, it depended on pH value of the immersing solution. On the other hand, the ejection of Na^+^ ions showed a diffusion-controlled release with two release phases in both solutions.

Figure 3c, d presents the cumulative concentrations of Al^3+^ coming out from both aluminosilicate frameworks (ZA and ZX). The contents of Al^3+^ in solutions incubated ZSM-5 were out of the instrumental detection limit and are omitted from the curve. The absence of aluminum ions is strongly supported by the Al-poverty of the parent zeolite composition. In contrast to the sodium manner, the release profiles of Al^3+^ in FaSSGF showed a significant difference from that of FeSSGF. Two stages were marking the evolution of aluminum ions in containing media with no significant difference for the detected amounts of Al^3+^ for each zeolite, either in FaSSGF or in FeSSGF. However, the solution pH seemed to be the limiting factor in controlling the Al^3+^ ion detachment from zeolites.

There were significant differences (*p* < 0.01) between the amounts of Al^3+^ released into FaSSGF and that in FeSSGF. These differences can be observed clearly from the calculated rates of eluted aluminum during stages I and II. For instance, the measured amounts of deliberated Al^3+^ were 37.4 and 33.5 ppm h^−0.5^ in FaSSGF, and 15.6 and 21.7 ppm h^−0.5^ in FeSSGF for ZA and ZX, respectively. Meanwhile, during stage II, the corresponding rates were 23.9 and 19.3 ppm h^−0.5^ in FaSSGF, and 1.4 and 0.9 ppm h^−0.5^ in FeSSGF, respectively. Likewise, the release of Al^3+^ ions showed a diffusion-controlled release with two release phases in both solutions.

Similar to Al^3+^, the release profile of Si^4+^ ions in FaSSGF was also significantly different from that in FeSSGF as can be seen from Fig. 3e, f. Obviously, the amounts of Si^4+^ ions that flushed out from the aluminosilicate species were significantly (*p* < 0.0003 for FaSSGF and *p* < 0.008 for FeSSGF) higher than that came out from ZSM-5, while the differences of silicon quantities in both solutions were insignificant (*p* < 0.4). Moreover, the amount of detected silicon ions in FaSSGF was significantly (*p* < 0.008) higher than that measured in FeSSGF for the corresponding zeolite samples. In the previous figure, the release profiles of silicon ions for all zeolites were presented in two stages. The release profile of silicon cations out of zeolite samples was somewhat similar to that of aluminum ions detachment. On the other hand, Si^4+^ elution rates showed that the release in both stages occurred in two stages. Moreover, the release rates were higher in FaSSGF than that in FeSSGF during stages I and II; these behaviors were similar to the release behaviors of Al^3+^ ions. In the rapid release stage (stage I), the release rates of Si^4+^ ions from ZSM-5, ZA and ZX in FaSSGF were 0.3, 16.1 and 23.1 ppm h^−0.5^, and in FeSSGF were 0.73, 5.7 and 8.9 ppm h^−0.5^, respectively, while they demonstrated almost similar values during stage II which were 0.06, 3.6 and 2.5 ppm h^−0.5^ in FaSSGF, and 0.06, 3.7 and 2.5 ppm h^−0.5^ in FeSSGF, respectively. Similar to Al^3+^ ion release, the Si^4+^ ion release was shown a diffusion-controlled release with two release phases in both solutions.

#### Interpretation of zeolite ion release

To explain the release of zeolite ions (Na^+^, Al^3+^ and Si^4+^) in a given solution, the cation exchange capacity, known for zeolites (CEC), is one of the relevant options. This character is typically demonstrated in zeolites and dated back to their genesis. During formation, the isomorphous substitution of zeolite tetrahedral Si^4+^ by Al^3+^ (SiO_4_ tetrahedra is the main building unit of alumino silicate zeolite) occurred and negatively charged vacancies are to be initiated. Those (−ve) places are compensated by the introduction of some (+ve) cations from the solutions (called exchangeable cations, EC), such as Na^+^, K^+^, Ca^2+^, and Mg^2+^. The number of EC is proportional to Al content of any aluminosilicate zeolite (Munthali et al. 2014; Kühl et al. 1999). Therefore, zeolite with lower Si/Al ratio (i.e., with high Al content) normally has high number of exchangeable cations. Accordingly, ZA with the least Si/Al ratio (Si/Al = 1.12) is presumed to have the highest amounts of Na^+^ and the highest CEC compared to ZX (Si/Al = 1.75) and ZSM-5 (Si/Al = 116.72). ZSM-5 composition has the least aluminum contents (0.4 per unit cell) among other structures and its effect on the framework dissolution can reasonably be neglected. Depending on this concept, ZA and ZX exhibited a high selectivity to hydronium ion (H^+^) than that of ZSM-5. H^+^ ions (ionic radius = 1.0 Å) originated from the dissociation of water and found their way to replace Na^+^ ions (ionic radius = 1.9 Å) in their framework sites (Munthali et al. 2015). Thus, sodium ions were increasingly eluted with high amounts to the supernatant at the early stages of immersion (stage I). At longer times (stage II) and due to the Na^+^ ion release, zeolite frameworks might have become unstable, especially in such high acidic solution, and suffered structural alteration with partial decay in the form of some silicon and aluminum cation evolution into the media. Under such conditions, the combined interaction of released Na^+^ ions with the dissociated Si and Al were likely to precipitate an amorphous layer of sodium aluminosilicate on zeolite surfaces to protect and prevent more Na^+^ ion release from zeolite assemblies and reached the steady-state release (Munthali et al. 2015; Nagy et al. 2011; Beran and Dubsky 1979). The whole zeolite dissolution profile can be seen in the light of the instability of their aluminosilicate structure in acidic media (Kuronen et al. 2000; Wilkin and Barnes 1998). In such highly acidic solution, FaSSGF (pH 1.6), an aggressive attack of the solution affected the zeolite structure greatly by preventing it from retaining its sodium contents. The release of weakly linked Na^+^ ions (van der Waals bond) from zeolite pores marked the beginning of the structure alteration at the first stage of immersion, and Na^+^ was replaced by H^+^ from water dissociation (Munthali et al. 2015). The release of sodium was followed by framework instability and subsequent release of Al^3+^ ions from the aluminosilicate zeolite (after 12 h), since Al^3+^ was more susceptible to acidic media than Si^4+^. At this stage, zeolite construction was then completely altered and its stability was deteriorated to the extent that the whole entity was subjected to extensive break down of its main building SiO_4_ units, which then started to be released into the solution in an amorphous phase. Comparatively, in less acidic medium, FeSSGF (pH5), Si^4+^ ion release might be due to Si–O–Si bonds breaking by water to form Si–OH groups, which in turn precipitated the dissolved silica again on zeolite surface as amorphous layer (Čimek et al. 1997). Therefore, this reaction did not depend mainly on Si-to-Al ratio, but it depended on the degree of oxygen bond breaking and hydration reaction.

The dissolution of zeolite main structure not only depends on the Si/Al ratio, but also depends on the particle size distribution of the zeolite grains, since the larger the particle, the higher will be its capabilities of withstanding the acidic media of the gastric fluids. Accordingly, Faujasite-NaX with the smallest grain size (2–3 µm) will have the largest surface area of contact with the attacking solution, resulting in more affinity toward structural dissolution. Meanwhile, the case of zeolite-A dissolution may be due to its low Si/Al content, combined with its smaller grain size of 5–6 µm. In the same context, the higher stability of ZSM-5 and its ability in resisting decay in the acidic gastric conditions are attributed to its high Si/Al ratio, along with the relatively higher sizes of its particles (6–7 µm).

Taking into consideration the small sizes of the used zeolites, along with their low Si/Al contents, combined with the long incubation period of 96 h and the detachment of a very small amounts of the main backbone structural elements (Si^4+^ and Al^3+^), compared to Na^+^ (naturally of exchanging nature), zeolites can be considered as a material with relevant stability for carrying and delivering drugs even in highly acidic media such as FaSSGF.

For avoiding the problem of aluminum loss in the organism, grain size of zeolite particle can be increased to ≥ 7 µm (the highest grain size in the current study) to enable the close-fitting structure of zeolite crystal to withstand the aggressive acidity. The idea, during mineral synthesis, is especially from raw materials of layered structure such as kaolin rock in our case, the born crystals used to attach to parent raw sheets, which leave the chances for the presence of part of rock debris of amorphous structure and easy decay nature. Thus, zeolites with complete crystallinity, higher grain sizes ≥ 7 µm and high Si/Al ratios were considered of more stability and safety in use for the drug delivery purposes.

### Drug delivery test

#### Drug release profiles

**Fig. 4:**
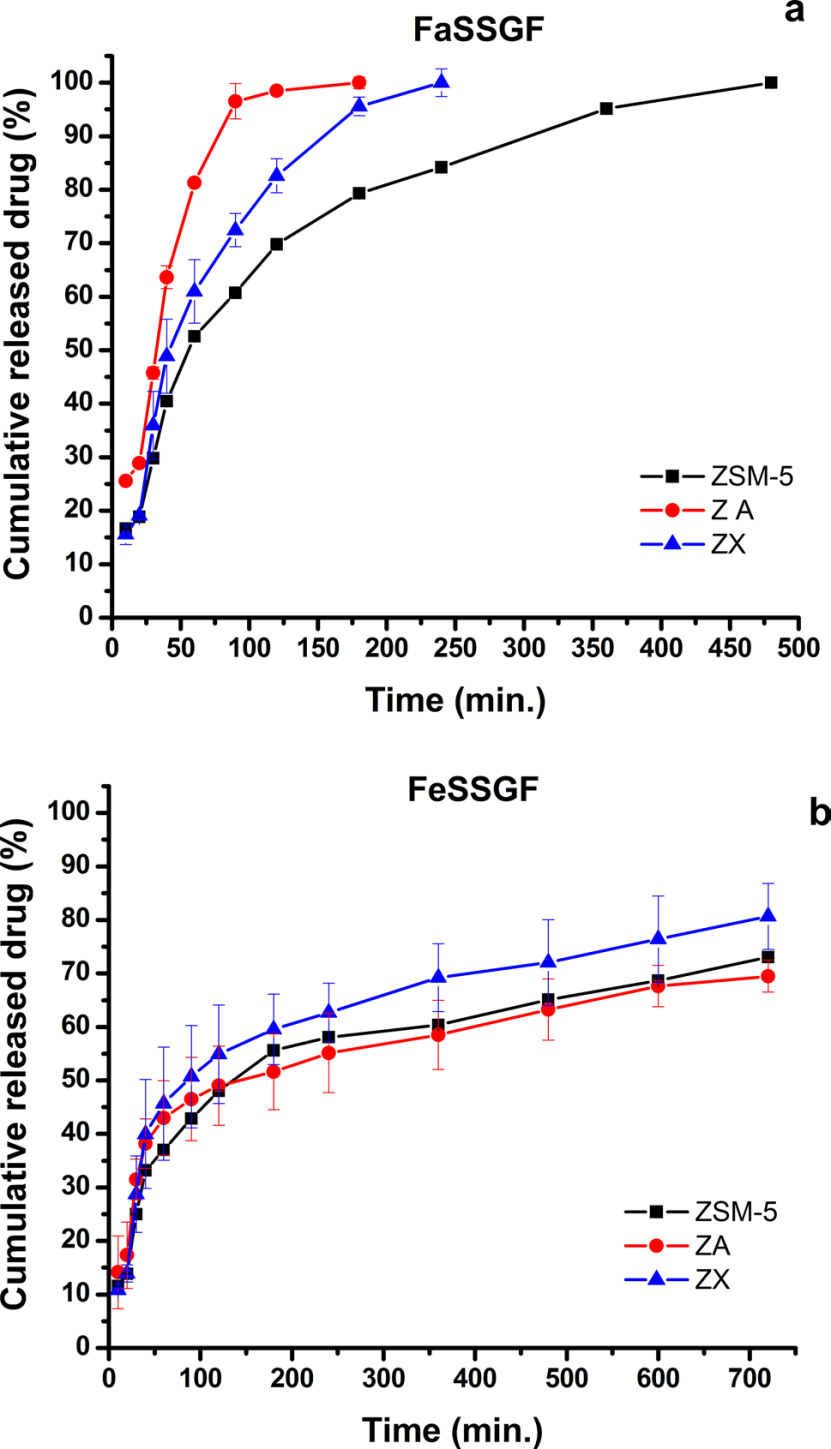
Cumulative 5-Fu release (%) from ZSM-5, ZA and ZX in distilled FaSSGF and FeSSGF

The percentages of drug hosted in zeolite samples were 77.9 ± 0.4%, 70.8 ± 0.8% and 71.5 ± 0.7% for ZSM-5, ZA and ZX, respectively. The release profile of 5-Fu from zeolite samples was investigated by studying the in vitro release in FaSSGF (pH 1.6) and FeSSGF (pH 5) at 37 °C (to simulate pH value of stomach and colon in the body). FeSSGF was prepared without milk addition because of the difficulty encountered in measuring the concentration of the released drug by UV–Vis spectroscopy due to the spectrophotometric interference arising from the opaque white color of milk. Figure 4a, b compares the in vitro 5-Fu released from ZSM-5, ZA, and ZX samples in FaSSGF and FeSSGF over a period of 12 h (720 min). In general, the drug found its way into the media by two-stage sustained release process; the first stage was rapid in which the drug was rushed into the solution (from 10 to 60 min), whereas, in the second stage, the slow release was dominant (from 60 to 720 min). However, in some samples, the drug was completely flushed out before the end of the soaking time (from 0 to 10 min). In FaSSGF, the Fu-5 was totally eluted within 480, 120 and 240 min from ZSM-5, ZA and ZX, respectively. Whereas, the encapsulated Fu-5 into zeolite pores and channels in the FeSSGF solution continued to release out during the whole span of incubation with total cumulative drug percentages of 73%, 70% and 81% for ZSM-5 ZA and ZX, respectively. Accordingly, the flushed Fu-5 percentages from the corresponding zeolite samples in FaSSGF were significantly (*p* < 0.05) higher than that measured in the FeSSGF. Hence, the release of 5-Fu by zeolites was pH dependent. Nevertheless, it was observed for FaSSGF and FeSSGF that the cumulative drug percentages released from zeolite ZSM-5 were almost lower than that released from ZA and ZX.

The release behavior of 5-Fu from the zeolites can be explained from different sides of view. It was possible to explain it from the charge and isoelectric point (IEP) of 5-Fu and zeolites. IEP of 5-Fu is 8.0 (Wenande et al. 2017), and so, it possesses a positive charge in aqueous media of pH < 8. Therefore, 5-Fu acquired a positive charge in FaSSGF (pH 1.6) and FeSSGF (pH 5). As mentioned before, aluminosilicate zeolites are usually synthesized by the substitution of zeolite tetrahedral Si^4+^ by Al^3+^, leaving behind negatively charged vacancies in zeolite structures which usually compensated with positively charged sodium ions, and consequently, the number of introduced Na^+^ is proportional to the number of substituted Si^4+^ by Al^3+^. In both simulated gastric fluids, as a result of Na^+^ ion leaching, zeolites have negative lattice charges, and these positions increased for more aluminous zeolites (Perminova et al. 2005). In this study, ZA and ZX possessed higher Si/Al ratio (1.12 and 1.75, respectively) than ZSM-5, thus contained a higher lattice negative charged places in the aqueous medium. Considering the IEP of 5-Fu, and taking zeolite Si/Al ratio into account, it can be stated that a relatively higher electrostatic attraction force was found between 5-Fu, and ZA and ZX than that generated between the drug and ZSM-5. Furthermore, as previously mentioned, silanol groups (Si–OH) were likely to be predominantly formed on ZSM-5 surfaces in aqueous solution due to breaking of Si–O–Si bonds by water to form Si–OH groups (Čimek et al. 1997); these groups can initiate hydrogen bonding with 5-Fu molecules. Yet, the drug was likely attached to ZA and ZX by electrostatic force, and by stronger hydrogen bonding for ZSM-5. Consequently, the drug liberated from ZSM-5 was slowed down.

The drug release profile can also be interpreted through the dissolution behavior of zeolites in the two simulated gastric fluids. The drug release profile was likely dependent on the whole zeolite dissolution profile. As mentioned before, the instability of zeolite was related to the destruction of aluminosilicate structure in the media (Kuronen et al. 2000; Wilkin and Barnes 1998), where the aluminosilicate structure was easier to be altered and decomposed in more acidic solution (FaSSGF) than in less acidic solution (FeSSGF); thus, the drug was released easier. Beside this description, 5-Fu was found to have higher solubility in more acidic media (El-Sherbiny et al. 2005). Therefore, the combined high zeolite dissolution and high 5-Fu solubility in acidic media explained the complete drug evolution in FaSSGF.

#### Drug release kinetics

To analyze the in vitro release kinetics of 5-Fu drug, different release data were fitted in different kinetic models (Higuchi and Baker–Lonsdale), and the correlation coefficient, *R*^2^, was used as indication of data fitting to know the mechanism of drug release from these formulations. Table 4 shows the release constants and regression coefficients (*R*^2^) as a result of the data fittings with Higuchi and Baker–Lonsdale kinetic models (the fitting curves are not shown). From the table, it was observed that the fitting of the data of the drug released in FaSSGF with Higuchi model demonstrated a high degree of linearity of one-stage drug release from ZSM-5, ZA and ZX, and the estimated release rates were 5.3, 10.8 and 9.2 h^−0.5^ for ZSM-5, ZA and ZX, respectively. ZX was the best fitted sample with Higuchi model (*R*^2^ = 0.971), whereas, ZSM-5 was the worst (*R*^2^ = 0.945). However, the drug release examined in the FeSSGF was shown a two-stage release. Stage I was a slow release stage, and the derived rates were 7.0, 7.6 and 9.7 h^−0.5^ for ZSM-5, ZA and ZX, respectively, whereas, stage II was shown as a fast release stage and the anticipated rates were 1.7, 1.4 and 1.7 h^−0.5^ for ZSM-5, ZA and ZX, respectively. Accordingly, the drug release from different zeolites in both fluids was proportional to h^−0.5^. Nevertheless, the drug release from ZSM-5, ZA and ZX occurred by diffusion mechanism from three zeolites into FaSSGF in one stage, while it was released in two stages by diffusion mechanism. Likewise, the curves of the drug release in FeSSGF fitting with Higuchi model was almost similar to the curves produced by the fitting of cumulative Al^3+^ and Si^4+^ ion concentrations with the square root of time, and consequently, the mechanism of drug released from the zeolite samples was directly related to the solubility mechanism of these zeolites, which was a pH-dependent solution.

Furthermore, different zeolite samples under investigation represented good linearity with Baker–Lonsdale equation, where *R*^2^ ranged from 0.889 to 0.993. This denoted that the drug was released from spherical zeolite particles. Interestingly, regression coefficient of ZSM-5 sample was high for both solutions. This attributed to a spherical shape of ZSM-5 sample, as it was confirmed by SEM micrographs.

### In vitro cytotoxicity cell culture evaluation of zeolite samples

**Fig. 5:**
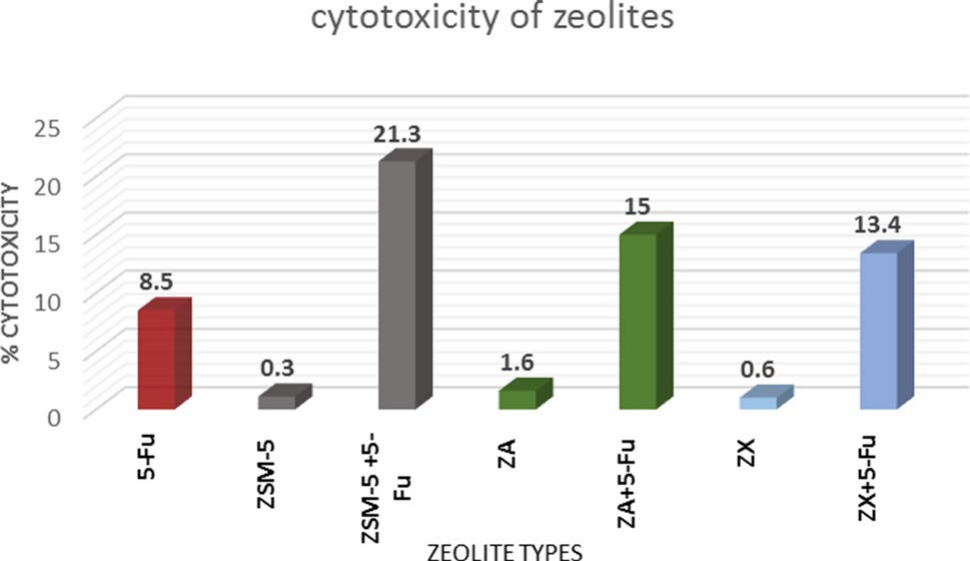
Cytotoxic effect of ZSM-5, ZA and ZX (100 µg/ml) with/without 5-FU on colon cancer cell line (CaCo-2) in comparison with pure 5-Fu using MTT assay (*n* = 3), data expressed as the mean value of cell cytotoxicity (% of control)

Inhibitory actions of each zeolite type and its minimum dose causing a harmful effect on the cells, as well as encapsulation of anticancer drug, 5-Fu, into the zeolite particles were investigated on the colon cancer cells (CaCo-2) using MTT assay after 48 h of incubation. Figure 5 shows the activities of free and drug-loaded zeolites in terms of cytotoxic percentage of lethal action after 48 h of incubation. Obviously, parent zeolites have neglecting percentages of cytotoxic effects; meanwhile, their drug-loaded versions showed appreciable cytotoxic action with the corresponding values of 21.3, 15, and 13.4% for ZSM-5, ZA, and ZX, respectively. Notably, ZSM-5 implied the lowest inhibition efficacy, followed by the other two low Si/Al zeolites. This may contradict the idea of the poisonous effect of Al released from zeolites, since ZSM-5 has nearly no aluminum contents compared to the other two zeolites ZA and ZX. Despite ZSM-5 had wider pore diameter than ZA and ZX, its structure stability was likely regulated by the sustained evolution of drug from the pores. Moreover, as previously mentioned, in the aqueous medium, 5-Fu molecules likely formed hydrogen bonding with the developed silanol groups on ZSM-5 surfaces, which slowed down the drug release from these zeolite particles and so decreased the drug cytotoxic effect on the cancer cells. On the other hand, silicon ions are known to enhance the cell viability due to their rapid adherence to the cell membranes (Absher and Mortara 1980).

**Fig. 6:**
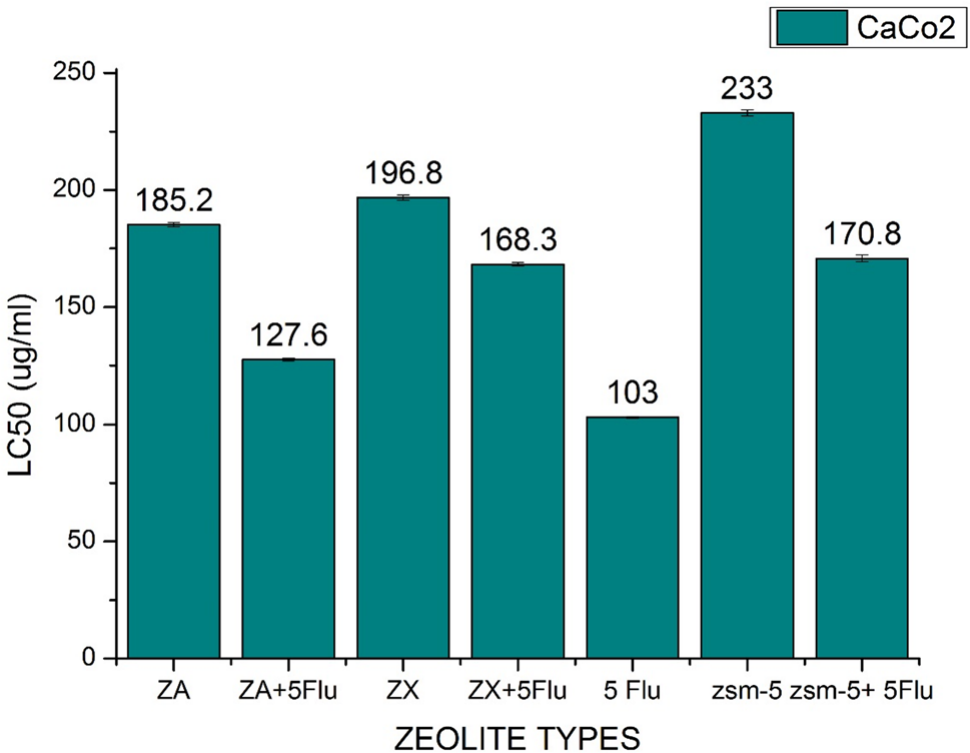
Cytotoxic effect (LC50 values) of free different zeolite samples (ZSM-5, ZA and ZX) and different zeolite samples conjugated with 5-Fu against colon cancer cell line (CaCo-2) compared to 5-Fu using MTT assay (*n* = 3), data expressed as the mean value of cell cytotoxicity (% of control)

Moreover, the cytotoxic effect of zeolite samples with and without 5-Fu on CaCo-2 cell line at different concentrations ranging from 100 to 12.5 µg/mL was screened to calculate LC50 value for each, as shown in Fig. 6. As shown in the figure, ZA was the most potent in free and loaded samples with 5-Fu followed by ZX and ZSM-5 with 127.6, 168.3 and 170.8 µg/mL, respectively, compared to drug-free samples (185.2, 196.8 and 233 µg/mL, for ZA, ZX and ZSM-5, respectively). 5-Fu is widely referred to as a chemotherapy drug against different solid tumors. However, 5-Fu has several limitations including short biological half-life due to rapid metabolism, inadequate and non-uniform oral absorption, harmful reactions on bone marrow and the gastrointestinal tract, and non-specific activity against solid cells (Li et al. 2008). In addition, there is evidence that it has an impact of standard-dose chemotherapy on cognitive function (Winocur et al. 2006). From the above results, the incorporation of 5-Fu with ZA, ZX, and ZSM-5 has successfully managed to decrease the 5-Fu dose by maintaining its efficacy as well, and it is expected to have more potent cytotoxic action on the viability of CaCo-2 cells at higher concentrations assay of more than 100 µg/mL.

## Conclusion

ZSM-5, zeolite A and faujasite-NaX zeolites (encoded, ZSM-5, ZA and ZX) were synthesized in this study mainly from cost-effective kaolin raw material using a low-energy microwave approach. The zeolite dissolution effect on the drug release behaviors was studied. The investigation of Na^+^, Al^3+^ and Si^4+^ ion release profiles of zeolites in two simulated gastric fluids (FaSSGF and FeSSGF) showed that the behavior of degradation and ion release were related to, or better said affecting the release profiles of 5-Fu from zeolite particles and the inhibition effect of 5-Fu-free zeolites and 5-Fu-conjugated zeolites on the cancer cells (CaCo-2). Similarity in many aspects, the ion liberation has occurred through two stages; rapid (1–12 h) and fast release (12–96 h), and the amounts of eluted ions were pH dependent, where they were significantly higher in fluid of pH 1.6 than in that of pH 5. Likewise, 5-Fu was released from different zeolites almost by a sustained release through two stages (rapid and steady-state release), as well as the percentage of released drug from the zeolite samples was pH dependent, where they were significantly higher in fluid of pH 1.6 than in that of pH 5. Moreover, the detachment of drug to zeolites was mainly dependent on the dissociation and ion release behaviors of zeolites. Finally, the zeolites have shown distinct antitumor activity against CaCo-2 cells when loaded with 5-Fu. Hence, these zeolites can be used as good reservoir for anticancer drug which releases in a controlled manner. All prepared zeolites showed a safe behavior for the CaCo-2 cells; meanwhile, all were of cytotoxic action when loaded with the 5-Fu drug.
